# Diagnostic value of whole-tumor ADC histogram parameters combined with ISUP grade and MRI-EPE score for predicting extracapsular extension in PI-RADS 4–5 prostate cancer

**DOI:** 10.3389/fonc.2026.1886143

**Published:** 2026-08-17

**Authors:** Lei Yi, Yue Gu, Xiaoliang Xie, Wei Wang, Xuncheng Yan, Yi Zhao

**Affiliations:** 1 Department of Radiology, The Affiliated Hospital of Yangzhou University, Yangzhou University, Yangzhou, Jiangsu, China; 2 Department of Radiology, Rugao People’s Hospital Affiliated to Kangda College of Nanjing Medical University, Rugao, Jiangsu, China

**Keywords:** apparent diffusion coefficient, extracapsular extension, histogram, magnetic resonance imaging, prostate cancer

## Abstract

**Purpose:**

To investigate the incremental value of whole-tumor apparent diffusion coefficient (ADC) histogram parameters, when combined with clinical pathological variables for predicting extracapsular extension (EPE) in patients with PI-RADS 4–5 prostate cancer.

**Methods:**

This retrospective study included 102 patients with pathologically confirmed prostate cancer following surgery, comprising 51 patients with EPE and 51 without EPE as the training cohort, plus an independent temporal validation cohort of 34 patients. All patients underwent preoperative multiparametric magnetic resonance imaging (mpMRI). Tumor regions and 1–3 mm peritumoral regions of interest (ROIs) were manually segmented on ADC maps using 3D Slicer software, followed by the extraction of histogram parameters. Histogram features were selected using forward stepwise logistic regression based on the likelihood ratio test. Clinical, histogram, and combined predictive models were established using logistic regression analysis. Diagnostic performance was assessed using receiver operating characteristic (ROC) curve analysis. Internal validation was performed with 1000 bootstrap resamples to evaluate model stability and optimism. Calibration and decision curve analyses were further applied to assess model accuracy and clinical utility. To specifically quantify incremental value beyond the established clinical model, continuous net reclassification improvement (NRI) and integrated discrimination improvement (IDI) were defined as core secondary endpoints and calculated in the temporal validation cohort.

**Results:**

Multivariate Firth-corrected logistic regression analysis identified the International Society of Urological Pathology (ISUP) grade (OR = 2.701, *p* = 0.033) and MRI-EPE score (OR = 5.441, *p* = 0.020) as independent clinical predictors of EPE. Among the single-region histogram models, the peri_1mm model achieved the highest diagnostic performance (AUC = 0.839). Peri1_Minimum and peri3_Minimum were selected as the most informative histogram features for model construction. The combined model, incorporating ISUP grade, MRI-EPE score, peri1_Minimum, and peri3_Minimum, demonstrated the best overall performance with an AUC of 0.917, which was significantly higher than that of the histogram model alone (AUC = 0.858, *p* = 0.026), while showing no significant difference compared with the clinical model (AUC = 0.903, *p* = 0.336). Bootstrap internal validation yielded corrected AUC values of 0.904, 0.890, and 0.845 for the combined, clinical, and histogram models, respectively. The combined model also showed excellent calibration and provided greater net clinical benefit across a broader range of threshold probabilities on decision curve analysis. Notably, in the independent temporal validation cohort, although the combined model’s AUC (0.861) remained numerically higher than that of the clinical model (0.818, *p* = 0.208), the addition of peritumoral ADC histogram features resulted in a significant continuous NRI of 0.7286 (95% CI: 0.0971-1.3601, *p* = 0.024) and a borderline significant IDI of 0.0919 (*p* = 0.053), indicating that the addition of peritumoral ADC histogram features led to a net improvement in the direction of predicted risk changes, beyond what was provided by the clinical model alone. The combined model also showed good calibration, with a calibration slope of 0.710 (95% CI: 0.273–1.148) and a Brier score of 0.150.

**Conclusion:**

Integration of ISUP grade, MRI-EPE score, and peritumoral ADC histogram features (peri1_Minimum and peri3_Minimum) enables robust prediction of EPE in PI-RADS 4–5 prostate cancer. While the combined model’s overall discriminatory performance was comparable to that of the clinical model based on AUC, the significant continuous NRI suggests incremental value in refining risk stratification. However, given the non-significant AUC difference and borderline IDI, these findings warrant validation in larger, multicenter cohorts before informing individualized surgical planning for high-risk patients.

## 1 Introduction

Prostate cancer (PCa) is among the most common malignancies affecting men worldwide (1), and accurate clinical staging is critical for selecting appropriate individualized treatment strategies (2). Extracapsular extension (EPE) represents an important marker of local tumor progression, influencing both the feasibility of nerve-sparing radical prostatectomy and the risk of positive surgical margins (3). The Prostate Imaging Reporting and Data System version 2.1 (PI-RADS v2.1) was developed to standardize the acquisition and interpretation of multiparametric magnetic resonance imaging (mpMRI) (4). Lesions categorized as PI-RADS 4 or 5 are considered highly suspicious for clinically significant prostate cancer and generally require biopsy confirmation (5). However, conventional MRI assessment of EPE primarily depends on subjective evaluation of morphological features, which is associated with substantial interobserver variability and limited sensitivity for detecting microscopic invasion (6). Alessi et al. reported that PI-RADS scores of 1–3 have a high negative predictive value (NPV) of up to 98% for excluding EPE (≥ pT3a) (7). In comparison, PI-RADS 4–5 lesions exhibit considerable biological heterogeneity, and conventional imaging techniques are often unable to capture subtle microenvironmental changes associated with EPE, leading to uncertainty in clinical assessment.

The apparent diffusion coefficient (ADC), derived from diffusion-weighted imaging (DWI), quantitatively reflects the degree of water diffusion restriction within tissues (8). ADC histogram analysis allows quantitative assessment of intratumoral heterogeneity and has shown promise in evaluating prostate cancer aggressiveness (9–11). However, the diagnostic value of ADC histogram parameters in PI-RADS 4–5 lesions has not yet been fully established. The peritumoral region may better reflect tumor invasiveness and microenvironmental remodeling, including lymphovascular infiltration, angiogenesis, lipid alterations, and edema (12). Most previous ADC histogram studies have focused primarily on intratumoral features, while largely overlooking the dynamic interactions between tumors and their surrounding microenvironment that contribute to invasive behavior (13–17). Therefore, this study aimed to quantitatively evaluate ADC histogram characteristics in peritumoral regions at different radial distances and to determine their contribution to the diagnosis of EPE.

At present, no standardized definition exists for the peritumoral region of interest (ROI). A narrowly defined ROI may fail to capture critical peritumoral information, whereas an excessively broad ROI may introduce noise from adjacent normal tissues and dilute relevant imaging features (12). In a pathological study involving 371 radical prostatectomy specimens, Chao et al. (18) reported a median radial EPE distance of 2.4 mm among 121 EPE-positive cases, with only 19% extending beyond 4 mm. Further, Lin et al. (19) demonstrated that radiomic features extracted from a 3-mm peritumoral ROI independently predicted bone metastasis in PCa, providing a rationale for the ROI expansion strategy adopted in this study. Therefore, patients with PI-RADS 4–5 prostate cancer were enrolled and systematically analyzed for ADC histogram features within intratumoral and peritumoral ROIs (1, 2, and 3 mm) using whole-tumor three-dimensional segmentation. By integrating these imaging features with important clinicopathological variables, including ISUP grade, which reflects tumor differentiation and metastatic potential (20), and MRI-EPE score, which evaluates capsular disruption and neurovascular invasion (21), a multimodal combined diagnostic model was developed. The objective of this study was to identify objective imaging biomarkers that could improve EPE prediction and support individualized treatment planning for patients with high-risk prostate cancer.

## 2 Materials and methods

### 2.1 Study population

This retrospective study was approved by the local Institutional Review Board (IRB) (The Affiliated Hospital of Yangzhou University), and informed written consent was waived by the IRB. All methods were performed in accordance with the relevant guidelines and regulations.

This single-center retrospective study consecutively included patients with pathologically confirmed prostate cancer who underwent radical prostatectomy (RP) between January 2023 and December 2025 as the training cohort. Additionally, we collected data between January and June 2026 to serve as an independent temporal validation cohort. The inclusion criteria were: (1) availability of complete preoperative multiparametric MRI (mpMRI) examinations with diagnostic image quality; (2) a Prostate Imaging Reporting and Data System (PI-RADS) score of 4 or 5; (3) definitive confirmation of extracapsular extension (EPE) status based on postoperative pathological findings; (4) complete clinical, imaging, and pathological records; and (5) Lesions located in the peripheral zone, transition zone, or bilateral zones.

**Figure 1:**
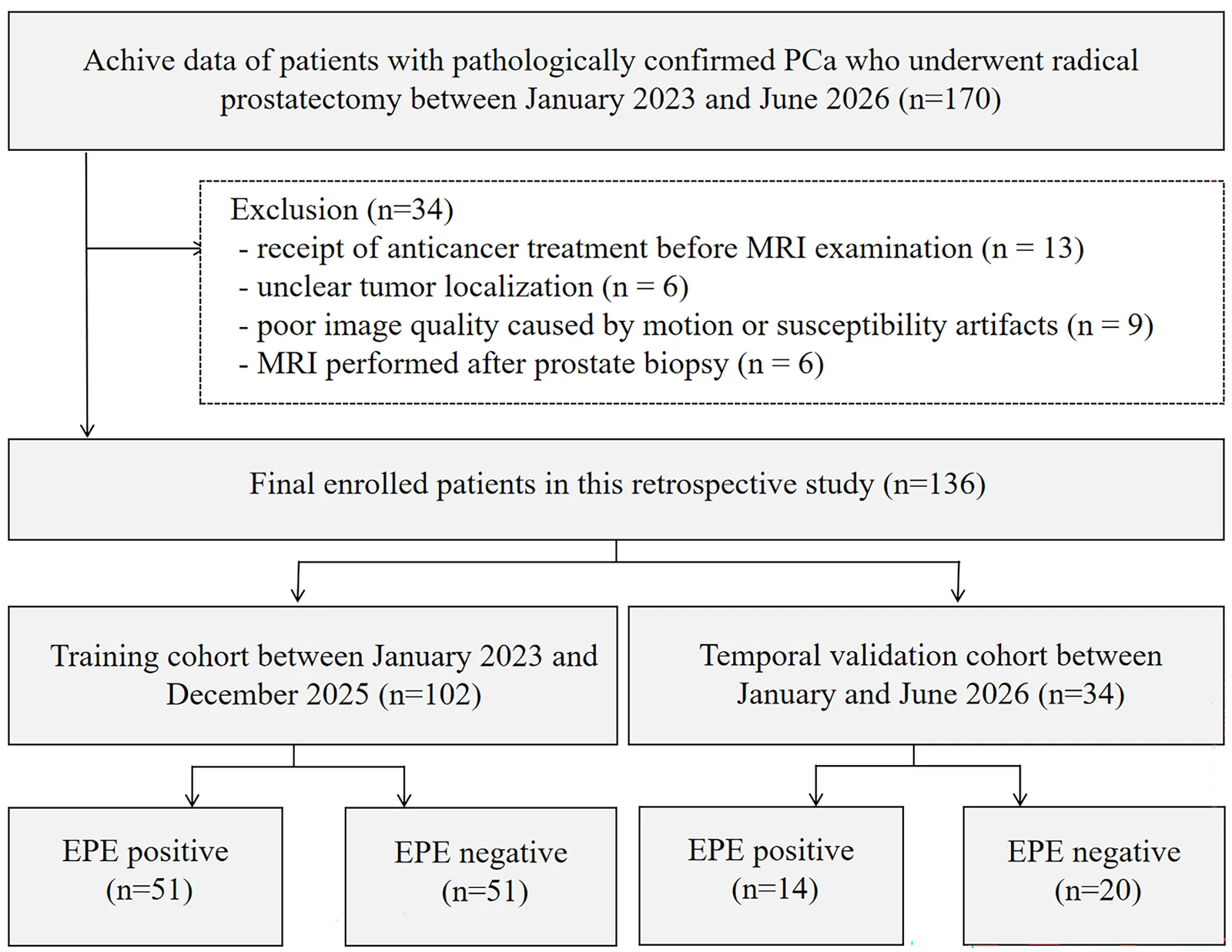
Flowchart for patient enrollment.

A total of 34 patients were excluded for the following reasons: receipt of additional anticancer treatment before MRI examination (n = 13), unclear tumor localization (n = 6), poor image quality caused by motion or susceptibility artifacts (n = 9), and MRI performed after prostate biopsy (n = 6). Ultimately, 136 patients were included in the final analysis, comprising 51 in the EPE-positive group and 51 in the EPE-negative group in the training cohort, and 14 in the EPE-positive group and 20 in the EPE-negative group in the temporal validation cohort. The prevalence of EPE was 47.79% (65/136). The process of patient enrollment is shown in **Figure 1**.

### 2.2 MRI acquisition protocol

All MRI examinations were conducted using a 3.0 T scanner (Architect 3.0T, GE Healthcare, Chicago, IL, USA) equipped with a 16-channel body phased-array coil and a 40-channel posterior coil. Patients were examined in the supine position with a moderately filled bladder. The imaging protocol consisted of axial T1-weighted imaging (T1WI), axial/coronal/sagittal T2-weighted imaging (T2WI), and axial diffusion-weighted imaging (DWI).

The main acquisition parameters were as follows: (1) axial T1WI: repetition time (TR)/echo time (TE) = 581/15.7 ms, slice thickness/interslice gap = 3.0/0 mm, field of view (FOV) = 200 × 200 mm, and number of excitations (NEX) = 1.5; (2) axial T2WI: TR/TE = 3263/107 ms, slice thickness/interslice gap = 3.0/0 mm, FOV = 200 × 200 mm, and NEX = 2.5; (3) coronal and sagittal T2WI: acquisition parameters were similar to those of axial T2WI, with adjustments made to the FOV as required; (4) DWI: TR/TE = 5215/85 ms, slice thickness/interslice gap = 3.0/1.0 mm, FOV = 260 × 260 mm, NEX = 16, and b-values of 0 and 2000 s/mm^2^. ADC maps were subsequently generated on the workstation using b-values of 0 and 2000 s/mm^2^.

### 2.3 Image segmentation and histogram feature extraction

ADC maps were imported into 3D Slicer software (version 5.10.0; 3D Slicer) for further analysis. To minimize intensity inhomogeneity, all images were preprocessed using the N4ITK MRI Bias Field Correction module. Voxel dimensions were subsequently resampled to 1×1×1 mm³. Image intensities were discretized using a fixed bin width of 32, and discretization using the bin width method yields more reproducible features (22–24).

A genitourinary radiologist, blinded to the pathological EPE status, performed semi-automatic segmentation of prostate cancer lesions on ADC maps with reference to T2-weighted imaging (T2WI) and diffusion-weighted imaging (DWI). Regions of interest (ROIs) were manually delineated slice-by-slice along the tumor margins. Peritumoral ROIs were generated using the margin expansion function, by dilating the tumor boundary outward by 1, 2, and 3 mm. Subsequently, manual refinement was performed to eradicate non-prostatic tissues, including periprostatic fat, seminal vesicles, rectum, urethra, and urinary bladder (22, 25).

Eighteen first-order histogram features were extracted from each ROI, including the intratumoral region and the 1-, 2-, and 3-mm peritumoral regions. These parameters included the 10th percentile, 90th percentile, energy, entropy, interquartile range, kurtosis, maximum, mean absolute deviation, mean, median, minimum, range, robust mean absolute deviation, root mean squared, skewness, total energy, uniformity, and variance. To evaluate interobserver reproducibility, a second physician independently repeated the segmentation and feature extraction procedures for 30 randomly selected cases two weeks later.

### 2.4 Clinical and pathological data collection

**Figure 2:**
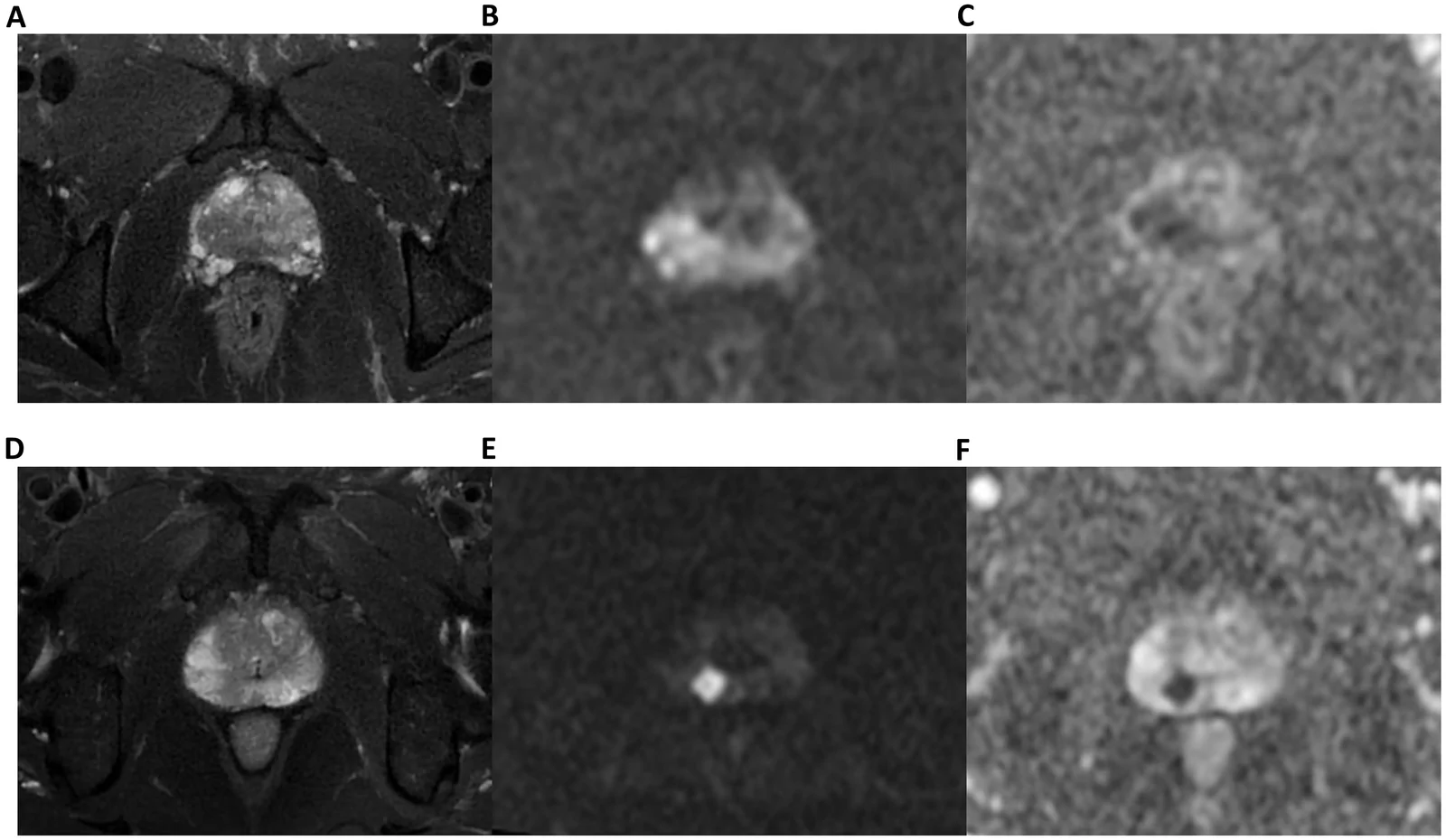
A 65-year-old male with EPE-positive prostate cancer.​ The right peripheral zone lesion exhibits low signal intensity on T2WI **(A)**, high signal intensity on DWI **(B)**, and corresponding low signal intensity on ADC map **(C)**. The lesion was assigned a PI-RADS score of 5 and an MRI-EPE score of 2. A 76-year-old male with EPE-negative prostate cancer. The right peripheral zone lesion exhibits low signal intensity on T2WI **(D)**, high signal intensity on DWI **(E)**, and corresponding low signal intensity on ADC map **(F)**. The lesion was assigned a PI-RADS score of 5 and an MRI-EPE score of 0.

Baseline clinical information was collected for all patients, including age, total prostate-specific antigen (tPSA), free PSA (fPSA), prostate volume, PSA density (PSAD), maximum lesion diameter, lesion location (peripheral zone, transitional zone, or bilateral involvement), PI-RADS score, and MRI-EPE score. The MRI-EPE score was independently assessed by two genitourinary radiologists with 7 and 9 years of experience in prostate MRI, respectively. Both readers were completely blinded to the final pathological EPE status. The readers were also blinded to each other’s scores throughout the assessment process. In cases where the two readers’ scores differed, a senior radiologist with 20 years of experience in prostate MRI, who was also blinded to pathological EPE status and the readers’ initial scores, reviewed all available imaging sequences and rendered the final consensus score. The final score was used for all subsequent analyses. Interobserver agreement between the two readers was evaluated using the intraclass correlation coefficient (ICC) with a two-way random-effects model for absolute agreement. The MRI-EPE was applied to evaluate preoperative multiparametric MRI (mpMRI) findings, as previously described (26) (**Figure 2**). MRI-EPE grading system: Grade 0, no capsular contact or capsular contact length (CCL) <15 mm; Grade 1, CCL ≥15 mm or capsule irregularity/bulging; Grade 2, CCL ≥15 mm and capsule irregularity/bulging; Grade 3, definite EPE with invasion into adjacent structures. The CCL was measured along the interface between the tumor and the prostatic capsule. All radical prostatectomy specimens were independently evaluated by a genitourinary pathologist with 11 years of experience. EPE positivity was defined as the microscopic extension of tumor cells beyond the prostatic fibromuscular stroma into the periprostatic adipose tissue or adjacent structures.

In patients with multiple lesions, the index lesion was defined as the lesion with the highest Gleason score or, when Gleason scores were identical, the largest-volume lesion on MRI. Tumor aggressiveness was classified according to the International Society of Urological Pathology (ISUP) Grade Group system (GG 1–5) based on the 2019 ISUP consensus conference criteria (20). The ISUP grade was determined from preoperative prostate biopsy specimens by an experienced genitourinary pathologist, and this information was available preoperatively for all patients.

### 2.5 Feature selection and model construction

Histogram features were standardized using Z-score normalization before analysis. Forward stepwise logistic regression based on the likelihood ratio test was performed to identify independent risk factors across five ROI categories: the intratumoral region, 1-mm peritumoral region, 2-mm peritumoral region, 3-mm peritumoral region, and the combined tumor-plus-peritumoral region. Corresponding histogram-based predictive models were subsequently established.

**Figure 3:**
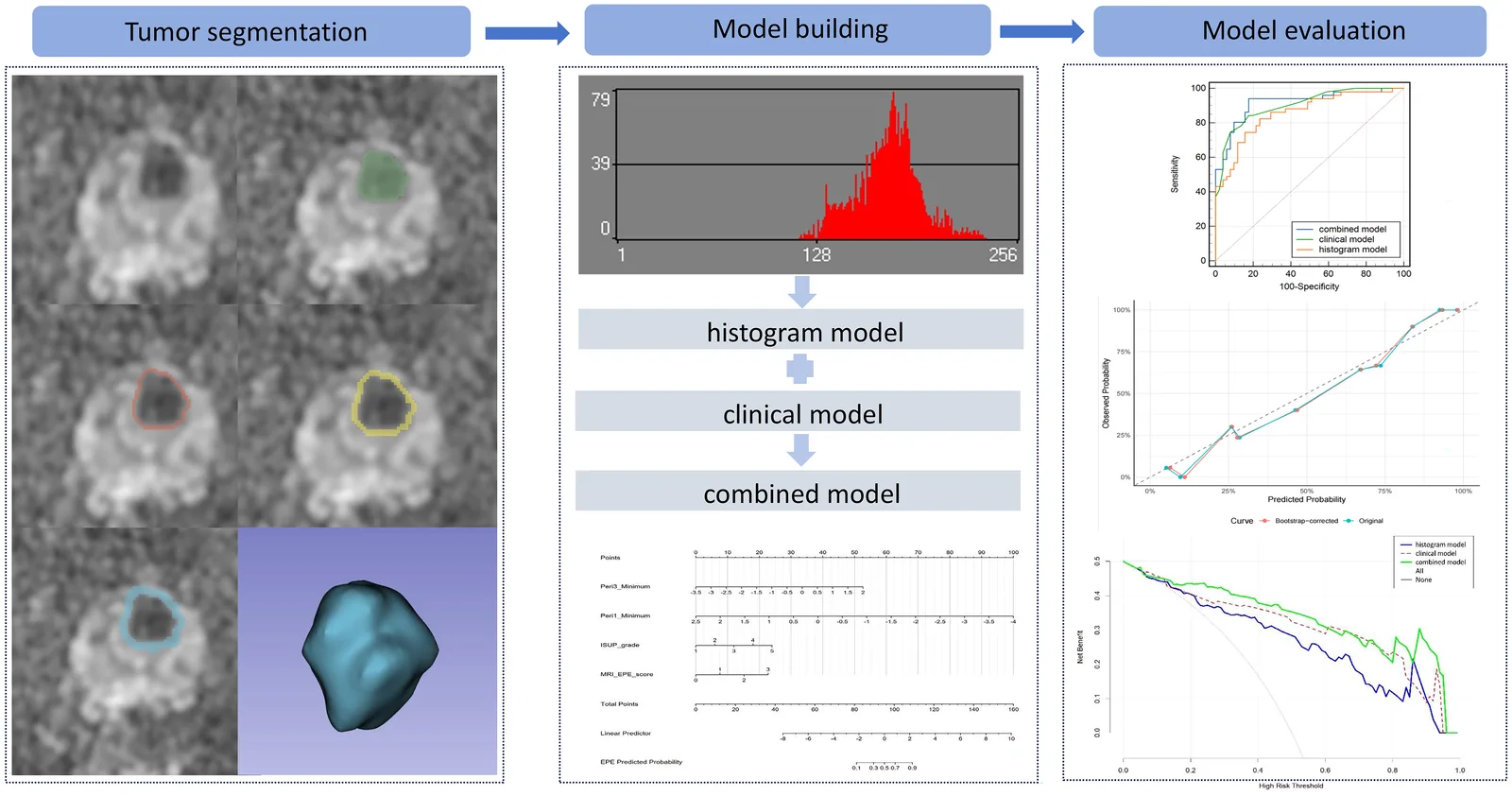
The workflow chart of this study.

Variables with *p* < 0.05 in the univariate analysis were entered into a multivariate Firth-corrected logistic regression to identify significant predictors and construct the clinical model. The predicted probabilities generated from the clinical and histogram models were then used as new variables in a subsequent logistic regression analysis to develop the combined model. The overall study workflow is presented in **Figure 3**.

The MRI-EPE score and ADC histogram features capture different imaging phenotypes. While the MRI-EPE score reflects qualitative morphologic evaluation by radiologists, ADC metrics provide quantitative, objective data via first-order statistics. Therefore, the subjective MRI-EPE score informed the clinical model, while the objective ADC features were allocated to the histogram model.

### 2.6 Statistical analysis

All statistical analyses were conducted using R software (version 4.2.0), MedCalc (version 22.0), and SPSS (version 26.0). Data normality was evaluated using the Shapiro–Wilk test. Normally distributed continuous variables were presented as mean ± standard deviation (SD) and compared using the independent-samples t-test. In comparison, non-normally distributed variables were expressed as median and interquartile range (IQR) and analyzed using the Mann–Whitney U test. Categorical variables were reported as frequencies and percentages [n (%)] and compared using the χ^2^ test.

Multicollinearity among variables was assessed using variance inflation factors (VIFs). Receiver operating characteristic (ROC) curve analysis was performed to determine the area under the curve (AUC), and differences between ROC curves were compared using DeLong’s test. Internal validation was performed using the bootstrap method with 1000 resampling iterations, and model calibration was evaluated using calibration curves. Decision curve analysis (DCA) was also performed to assess clinical utility. All statistical tests were two-sided, and *p*-values < 0.05 were considered statistically significant.

To interpret the combined model, SHAP (SHapley Additive exPlanations) analysis was performed using the fastshap package in R. For the linear logistic regression model, exact Linear SHAP values were obtained by setting exact = TRUE. For each prediction, the SHAP value of a feature represents its contribution to the log-odds of the prediction relative to the baseline (average prediction of the background dataset). However, since the model’s final output is transformed from log-odds to predicted probability via the logistic sigmoid function, these contributions directly translate to changes in the predicted probability of EPE. Feature importance was ranked by the mean absolute SHAP value across all patients. Visualization was performed using the shapviz package. To evaluate the incremental value of the combined model over the clinical model, reclassification analyses were performed in the temporal validation cohort. The continuous Net Reclassification Improvement (NRI) and Integrated Discrimination Improvement (IDI) were calculated using the reclassification function in the PredictABEL package. Calibration assessment employed four metrics: slope (β), calibration-in-the-large (α), O/E ratio, and Brier score. The slope β was estimated by logistic regression of observed outcomes on logit(p̂) (ideal β = 1). The intercept α was derived from a logistic model with slope fixed to 1 (offset term; ideal α = 0). The O/E ratio was calculated as ΣY/Σp̂ (ideal = 1). All metrics are presented with 95% confidence intervals (CIs).

## 3 Results

### 3.1 Patient demographics and tumor characteristics

A total of 102 patients were included in the study and divided equally into EPE-positive (n = 51) and EPE-negative (n = 51) groups (**Table 1**) in the training cohort. Compared with the EPE-negative group, patients in the EPE-positive group were significantly older (69.96 ± 6.27 vs. 73.25 ± 6.99 years, *p* = 0.014) and had larger lesions (median diameter: 3.10 vs 1.60 cm, *p* < 0.001). The EPE-positive group also demonstrated significantly higher median levels of total PSA (tPSA), free PSA (fPSA), and PSA density (PSAD), with values of 41.39, 4.74, and 1.00, respectively, compared with 15.15, 1.45, and 0.30 in the EPE-negative group (all *p* < 0.001).

**Table 1:** Baseline data and tumor MRI characteristics between groups in the training cohort.

Characteristics	EPE- (n=51)	EPE+ (n=51)	*p* value
age (year)	69.96 ± 6.27	73.25 ± 6.99	**0.014** ^a^
maximum diameter (cm)	1.60 (1.20, 2.50)	3.10 (2.00, 3.70)	**<0.001** ^b^
tPSA(ng/ml)	15.15 (7.27, 37.71)	41.39 (19.10, 84.11)	**<0.001** ^b^
fPSA(ng/ml)	1.45 (0.89, 4.54)	4.74 (2.41, 14.06)	**<0.001** ^b^
volume (cm3)	40.00 (28.28, 55.10)	42.00 (32.00, 55.20)	0.538^b^
PSAD (ng/mL/cm3)	0.30 (0.20, 0.56)	1.00 (0.50, 2.50)	**<0.001** ^b^
ISUP grade n (%)			<0.001^c^
1	7 (13.73)	0 (0.00)	
2	15 (29.41)	6 (11.76)	
3	24 (47.06)	13 (25.49)	
4	4 (7.84)	11 (21.57)	
5	1 (1.96)	21 (41.18)	
Location n (%)			<0.001^c^
bilateral zone	3 (5.88)	18 (35.29)	
peripheral zone	32 (62.75)	30 (58.82)	
transition zone	16 (31.37)	3 (5.88)	
PI-RADS score n (%)			<0.001^c^
4	23 (45.10)	2 (3.92)	
5	28 (54.90)	49 (96.08)	
MRI-EPE score n (%)			<0.001^c^
0	18 (35.29)	1 (1.96)	
1	27 (52.94)	14 (27.45)	
2	5 (9.80)	20 (39.22)	
3	1 (1.96)	16 (31.37)	

EPE-, EPE-negative; EPE+, EPE-positive.

^a^ Comparisons were performed by independent samples t-test.

^b^ Comparisons were performed by Mann−Whitney U test.

^c^ Comparisons were performed by χ^2^ test; p values in bold denote statistical significance.

With respect to pathological grading, ISUP grade groups 4 and 5 were substantially more common in the EPE-positive group, accounting for 21.57% and 41.18% of cases, respectively, whereas only 7.84% and 1.96% of patients in the EPE-negative group were classified as ISUP grades 4 and 5 (*p* < 0.001). Regarding lesion distribution, EPE-positive tumors were predominantly located in the bilateral and peripheral zones, whereas EPE-negative tumors were more frequently observed in the peripheral and transitional zones (*p* < 0.001).

Imaging findings further showed that PI-RADS 5 lesions were significantly more prevalent in the EPE-positive group than in the EPE-negative group (96.08% vs. 54.90%, p < 0.001). Similarly, higher MRI-EPE scores (2, 3) were observed more frequently in EPE-positive patients (70.59% vs 11.76%, *p* < 0.001). The MRI-EPE score demonstrated good inter-rater reliability, with an ICC of 0.798 (95% CI: 0.712–0.862, *p* < 0.001). No significant difference in prostate volume was identified between the two groups (*p* = 0.538). Additionally, the intergroup comparisons of baseline clinical characteristics in the temporal validation cohort are summarized in **Supplementary Table 1**.

### 3.2 Screening of independent predictive factors for clinical features

Multicollinearity analysis was performed using variance inflation factors (VIFs), and all VIFs were below 10, indicating no significant multicollinearity among the included variables. Multivariate Firth-corrected logistic regression analysis identified ISUP grade and MRI-EPE score as independent predictors of EPE (*p* = 0.033 and *p* = 0.020, respectively). The overall model demonstrated good fit according to the likelihood ratio test (χ^2^ = 58.70, *p* < 0.001).

ISUP grade and MRI-EPE score were subsequently incorporated as continuous variables to establish the clinical prediction model. The analysis showed that each one-grade increase in ISUP grade was associated with a 2.701-fold increase in the risk of EPE (adjusted OR = 2.701, 95% CI: 1.512–4.826). Similarly, each one-grade increase in MRI-EPE score corresponded to a 5.441-fold increase in EPE risk (adjusted OR = 5.441, 95% CI: 2.355–12.573).

### 3.3 Histogram feature screening and comparison

Interobserver reproducibility of histogram feature extraction was evaluated using intraclass correlation coefficients (ICCs), which ranged from 0.754 to 0.971, demonstrating good agreement between the two physicians. Forward stepwise logistic regression analysis identified two significant histogram features from each of the four analyzed regions, including the intratumoral region and the 1-, 2-, and 3-mm peritumoral regions, with 18 parameters evaluated per region. In total, eight significant predictors were obtained, as summarized in **Table 2**. Among these features, Peri1_Minimum and Peri3_Minimum were ultimately selected to establish the combined tumor-plus-peritumoral parameter set. All selected histogram features showed significant differences between the EPE-positive and EPE-negative groups (all *p* < 0.001). Additionally, the original ADC histogram parameters are provided in **Supplementary Table 3**.

**Table 2:** Comparison of histogram parameters of different regions of interest groups in the training cohort.

Segmentation region	Parameters	EPE- (n=51)	EPE+ (n=51)	*p* value
intratumor	Intra_90Percentile	0.45 ± 0.98	-0.45 ± 0.80	**<0.001** ^a^
	Intra_TotalEnergy	-0.56 (-0.66, -0.22)	-0.22 (-0.50, 0.65)	**<0.001** ^b^
peritumoral 1 mm	Peri1_Minimum	0.53 ± 0.84	-0.53 ± 0.86	**<0.001** ^a^
	Peri1_TotalEnergy	-0.56 (-0.69, -0.18)	-0.03 (-0.48, 0.71)	**<0.001** ^b^
peritumoral 2 mm	Peri2_Minimum	0.50 ± 0.92	-0.50 ± 0.82	**<0.001** ^a^
	Peri2_TotalEnergy	-0.60 (-0.74, -0.30)	0.00 (-0.48, 0.73)	**<0.001** ^b^
peritumoral 3 mm	Peri3_Minimum	0.61 (0.10, 1.06)	-0.40 (-0.97, 0.19)	**<0.001** ^b^
	Peri3_TotalEnergy	-0.65 (-0.75, -0.06)	-0.01 (-0.49, 0.87)	**<0.001** ^b^
Intra+Peri	Peri1_Minimum	0.53 ± 0.84	-0.53 ± 0.86	**<0.001** ^a^
	Peri3_Minimum	0.61 (0.10, 1.06)	-0.40 (-0.97, 0.19)	**<0.001** ^b^

ADC histogram parameters were presented as Z-score normalized values; Intra_, intratumoral; Intra+Peri, Intratumoral combined with peritumoral; Peri1_, peritumoral 1 mm; Peri2_, peritumoral 2 mm; Peri3_, peritumoral 3 mm.

^a^ Comparisons were performed by independent samples t-test.

^b^ Comparisons were performed by Mann−Whitney U test; p values in bold denote statistical significance.

### 3.4 Comparison of diagnostic efficacy of different parameters and models

Among the evaluated histogram features, Peri1_Minimum achieved the highest diagnostic performance (AUC = 0.825), whereas intratumoral TotalEnergy showed the lowest (AUC = 0.713). The AUC values for both Minimum and TotalEnergy progressively declined as the peritumoral expansion distance increased. Among the single-region histogram models, the peri_1mm model demonstrated the best diagnostic performance, with an AUC of 0.839. DeLong test analysis revealed a significant difference in AUC between the 1-mm and 3-mm peritumoral models (*p* = 0.021) (**Table 3**).

**Table 3:** Histogram parameters of different regions and model diagnostic efficacy in the training cohort.

Parameters and models	AUC (95% CI)	Sensitivity (%)	Specificity (%)
Intra_90Percentile	0.787 (95% CI 0.694, 0.862)	74.51	80.39
Intra_TotalEnergy	0.713 (95% CI 0.615, 0.798)	84.31	52.94
Peri1_Minimum	0.825 (95% CI 0.737, 0.893)	86.27	70.59
Peri1_TotalEnergy	0.778 (95% CI 0.663, 0.837)	88.24	54.90
Peri2_Minimum	0.802 (95% CI 0.712, 0.875)	78.43	72.55
Peri2_TotalEnergy	0.767 (95% CI 0.673, 0.845)	74.51	72.55
Peri3_Minimum	0.788 (95% CI 0.696, 0.863)	72.55	78.43
Peri3_TotalEnergy	0.753 (95% CI 0.658, 0.833)	82.35	64.71
intratumoral model	0.805 (95% CI 0.715, 0.877)	80.39	78.43
peri_1 mm model	0.839 (95% CI 0.753, 0.904)	78.43	80.39
peri_2 mm model	0.817 (95% CI 0.728, 0.886)	74.51	80.39
peri_3 mm model	0.794 (95% CI 0.702, 0.868)	82.35	68.63

**Figure 4:**
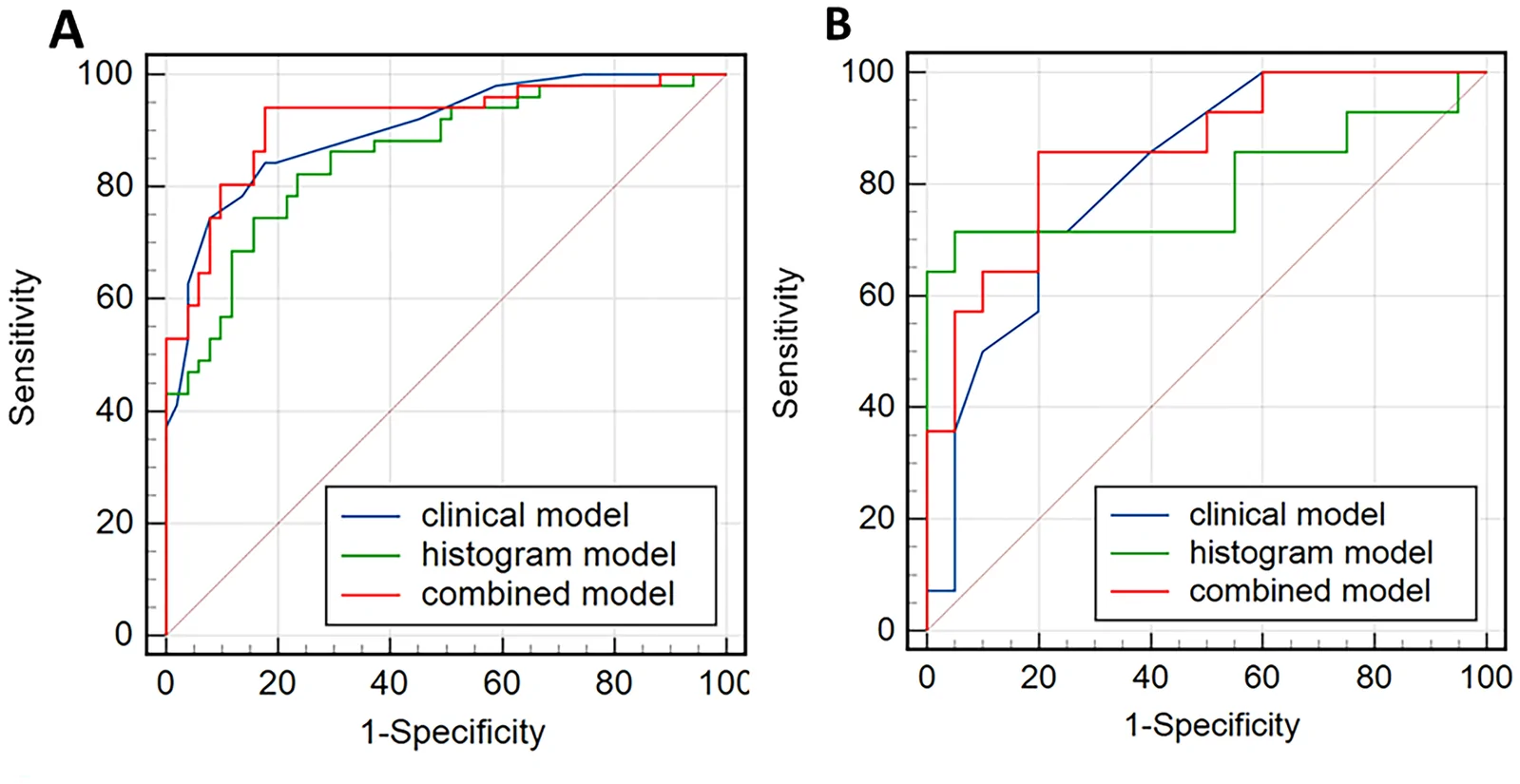
Receiver operating characteristic curves of different models in the training cohort **(A)** and independent temporal validation cohort **(B)**. In the training cohort, the combined model exhibited significantly higher AUC than the histogram model (*p*=0.026), while no significant differences were observed between the combined model and the clinical model (*p*=0.336) or between the clinical and histogram models (*p*=0.209). In the temporal validation cohort, no significant differences in AUC were found among any of the pairwise model comparisons (all *p*>0.05; combined vs. clinical: *p*=0.208; combined vs. histogram: *p*=0.383; clinical vs. histogram: *p*=0.833). *p*-values were calculated using the DeLong test for pairwise comparisons between models.

In the training cohort, for the histogram model, integration of the peri1_Minimum and peri3_Minimum features yielded an AUC of 0.858. The clinical model, constructed using ISUP grade and MRI-EPE score, achieved an AUC of 0.903. A combined model was subsequently developed by incorporating the outputs of both the clinical and histogram models into logistic regression analysis. This combined model demonstrated the highest diagnostic accuracy, with an AUC of 0.917. DeLong test results showed that the combined model significantly outperformed the histogram model alone (*p* = 0.026). In comparison, no statistically significant difference was observed between the combined and clinical models (*p* = 0.336) (**Table 4**; **Figure 4**). The regression coefficients, odds ratios, 95% confidence intervals, and p-values for each predictor in the clinical, histogram, and combined models are provided in **Supplementary Table 2**. The logistic regression equations for estimating the predicted probability of EPE were derived for each model. The clinical model was formulated as Logit(P) = −5.415 + 0.994 × ISUP grade + 1.694 × MRI-EPE score. The histogram model was expressed as Logit(P) = 0.010 − 4.209 × Peri1_Minimum + 2.398 × Peri3_Minimum. The combined model, which integrated the predicted probabilities from both the clinical and histogram models, was defined as Logit(P) = −3.716 + 4.547 × clinical model predicted probability + 2.983 × histogram model predicted probability.

**Table 4:** Comparison of diagnostic efficacy of different models. .

Cohort	Model	AUC (95% CI)	Sensitivity (%)	Specificity (%)
Training cohort	combined model	0.917 (95% CI 0.846, 0.962)	94.12	82.35
	clinical model	0.903 (95% CI 0.828, 0.953)	84.31	82.35
	histogram model	0.858 (95% CI 0.775, 0.919)	74.51	84.31
Temporal validation cohort	combined model	0.861 (95% CI 0.699, 0.955)	85.71	80.00
	clinical model	0.818 (95% CI 0.648, 0.929)	71.43	80.00
	histogram model	0.796 (95% CI 0.624, 0.915)	71.43	95.00

### 3.5 Internal validation and evaluation of the model

**Figure 5:**
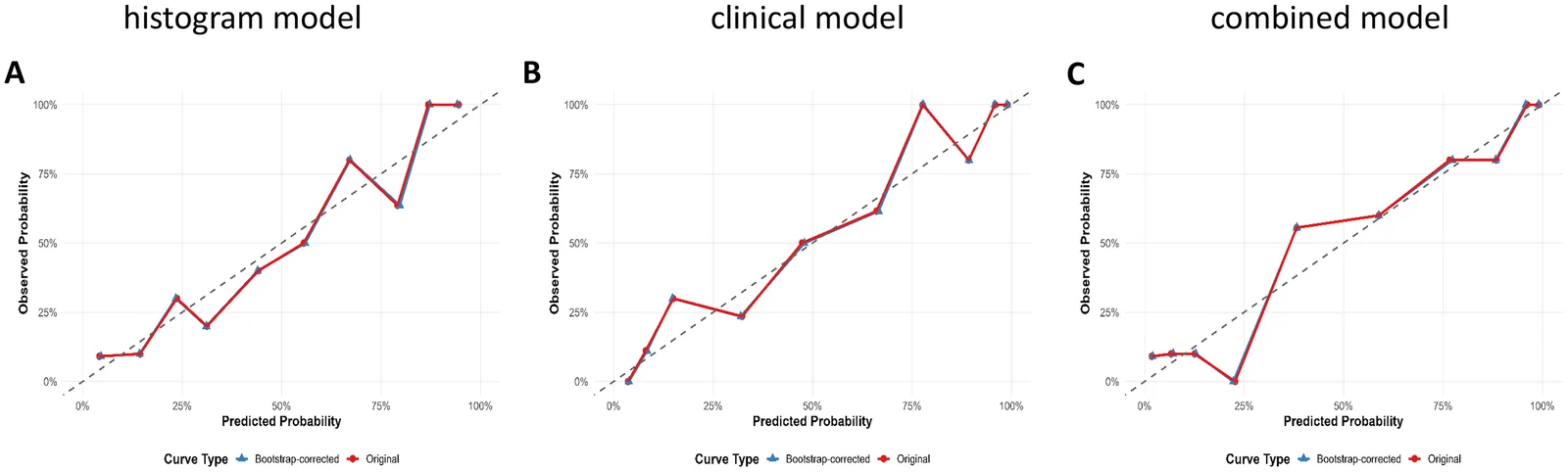
Calibration curves of the histogram model, clinical model, and combined model in the training cohort. The x-axis represents the predicted probability, while the y-axis indicates the observed probability. The dashed diagonal line serves as the reference line for perfect calibration. The red solid line depicts the original calibration curve, whereas the blue line represents the curve corrected by bootstrap resampling. A model with good calibration will have a curve that closely follows the diagonal dashed line, indicating minimal discrepancy between the model's predictions and the actual clinical outcomes.

Internal validation using the 1000-times bootstrap method demonstrated that the combined model achieved the strongest discriminative performance among all models (**Table 5**), with a corrected AUC of 0.904, exceeding those of both the clinical and histogram models. Regarding overall predictive accuracy, which reflects both discrimination and calibration, the combined model yielded the lowest corrected Brier score (0.126), representing reductions of 10.0% and 25.7% compared with the clinical model (0.140) and histogram model (0.170), respectively. The combined model showed the smallest mean absolute error (MAE; 0.223), indicating superior accuracy in individualized EPE risk prediction (**Figure 5**).

**Table 5:** Comparison of prediction performance based on 1000-time bootstrapping internal validation models in the training cohort.

Model	Adjusted AUC (95% CI)	Adjusted brier score	MAE
combined model	0.904 (95% CI 0.853, 0.959)	0.126	0.223
clinical model	0.890 (95% CI 0.839, 0.945)	0.140	0.249
histogram model	0.84 5(95% CI 0.794, 0.901)	0.170	0.312

MAE, Mean Absolute Error.

**Figure 6:**
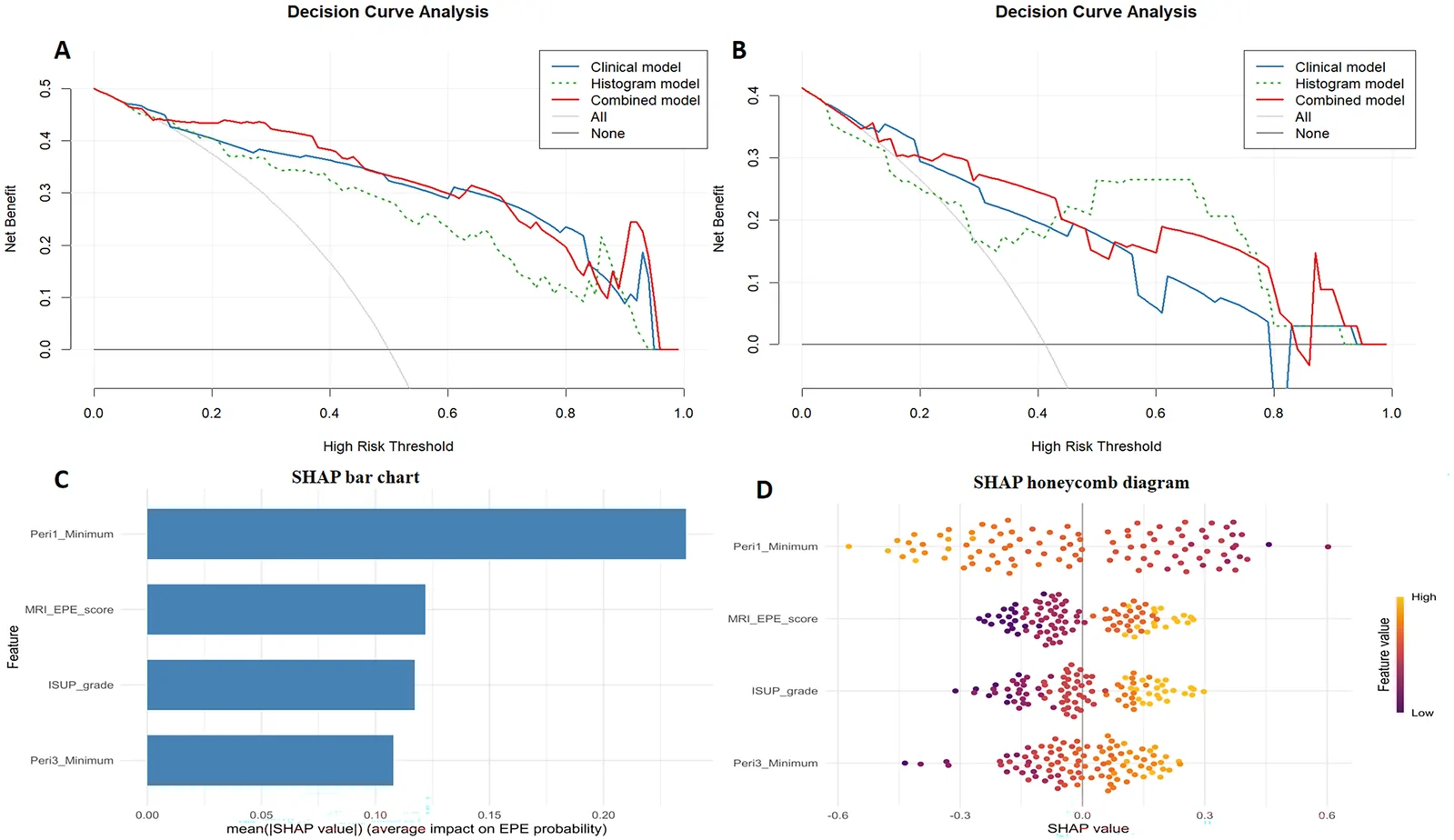
Model evaluation and visualization. **(A, B)** Decision curve analysis of the histogram model, the clinical model, and the combined model in the training cohort and temporal validation cohort. The y-axis represents the net benefit, and the x-axis represents the threshold probability. The combined model (red line) demonstrated higher net benefit than the clinical model (blue line) and the histogram model (green line) across a broad range of threshold probabilities in both cohorts, supporting its potential clinical utility. **(C)** SHAP bar chart illustrating the global feature importance derived from the SHapley Additive exPlanations values. The features are ranked by their mean absolute SHAP value, indicating their average contribution to the model's log-odds output, which corresponds to changes in the predicted probability of EPE. Peri1_Minimum contributed the most significantly to the model's predictions, followed by MRI_EPE_score, ISUP_grade, and Peri3_Minimum. **(D)** SHAP honeycomb diagram providing a local explanation of the model's behavior for individual predictions. Each dot represents a single SHAP value for a specific feature of an instance. The color gradient (purple to yellow) indicates the magnitude of the feature value from low to high. Features positioned to the right of the zero line (positive SHAP values) increase the predicted probability of EPE, while those on the left decrease it. This visualization shows how different feature values interact to produce a final risk prediction for a given patient.

Decision curve analysis (DCA) further demonstrated that the combined model provided greater clinical net benefit across a broader range of threshold probabilities than either the clinical or histogram models alone. SHAP analysis revealed that the relative contribution of features, ranked from highest to lowest, was Peri1_Minimum, MRI-EPE score, ISUP grade, and Peri3_Minimum (**Figure 6**). The nomogram derived from the combined model enabled intuitive visualization of individualized EPE prediction probabilities (**Supplementary Figure 1**).

### 3.6 Temporal validation and reclassification performance

To further validate the model’s generalizability, we applied the pre-established models to an independent temporal cohort (n = 34). In this validation cohort, the combined model achieved an AUC of 0.861, outperforming the clinical (0.818) and histogram (0.796) models, although the pairwise DeLong tests did not reach statistical significance (all *p* > 0.05).

Reclassification analyses were performed to evaluate the clinical incremental value. Compared with the clinical model, the combined model yielded a significant Continuous NRI of 0.7286 (95% CI: 0.0971-1.3601; *p* = 0.024), and the IDI was 0.0919 (*p* = 0.053), indicating that the addition of peritumoral ADC histogram features led to a net improvement in the direction of predicted risk changes. The calibration metrics for the three models are summarized in **Supplementary Table 4**. For all models, the 95% CIs of the slope, intercept, and O/E ratio included their ideal values (1, 0, and 1, respectively), indicating no statistically significant global miscalibration in the temporal validation cohort. The combined model showed the best calibration, with the lowest Brier score (0.150, 95% CI 0.057–0.243), a slope closest to 1 (0.710, 95% CI 0.273–1.148), and an O/E ratio (0.885, 95% CI 0.530–1.275) near unity. The clinical model had intermediate values (Brier = 0.161, slope = 0.682, O/E = 0.932). The histogram model exhibited the largest deviation, with the highest Brier score (0.203) and an O/E ratio (0.778) farthest from 1, suggesting a tendency toward over-prediction. These findings confirm that, beyond mere ranking ability (AUC), the combined model provides improvements in clinical risk reclassification.

## 4 Discussion

This study applied three-dimensional volumetric ADC histogram analysis to systematically investigate the diagnostic significance of intratumoral and peritumoral regions (1, 2, and 3 mm) for predicting EPE in PI-RADS 4–5 prostate cancer. Among all histogram parameters, peri1_Minimum demonstrated the highest diagnostic performance. Similarly, the peri_1mm model achieved the best performance among the regional models, highlighting the importance of quantitatively assessing the peritumoral microenvironment for EPE prediction. Peri1_Minimum and Peri3_Minimum were identified as the most informative histogram features, and the histogram model constructed using these parameters achieved an AUC of 0.858. After integrating the histogram model with ISUP grade and MRI-EPE score, the combined model’s diagnostic performance improved, reaching an AUC of 0.917. Bootstrap-corrected AUCs (0.904 vs. 0.890 vs. 0.845) supported the combined model’s robustness. While validation in a temporal cohort (n = 34) showed a maintained trend of superiority (AUC: 0.861), statistical significance was not met, likely due to cohort size. Notably, reclassification analysis revealed potential incremental value in risk reclassification compared with the clinical model, with the combined model achieving a continuous NRI of 0.7286 (*p* = 0.024). These results suggest that incorporating peritumoral ADC histograms enhances both diagnostic accuracy and clinical decision-making beyond conventional assessment.

In this study, patients in the EPE-positive group exhibited significantly higher PSA levels, larger tumor diameters, and a significantly higher proportion of PI-RADS 5 lesions, findings that are consistent with those reported by Wu et al. (27). Multivariate Firth-corrected logistic regression analysis further demonstrated that ISUP grade and MRI-EPE score were independent predictors of EPE. ISUP grade is a key pathological indicator of tumor aggressiveness and is closely associated with cellular differentiation, proliferative activity, and metastatic potential (20). The MRI-EPE score incorporates several morphological imaging features, including tumor-capsule contact length, capsular bulging, capsular disruption, and neurovascular bundle invasion. It is widely used in clinical imaging evaluation of EPE (21, 26). However, ISUP grade is routinely derived from preoperative prostate biopsy specimens and is readily accessible prior to surgical decision-making, it is inherently limited by unavoidable biopsy sampling error. The heterogeneous and multifocal nature of prostate cancer means that biopsies may lead to a discordance between biopsy-assigned ISUP grade and the final pathological ISUP grade determined from radical prostatectomy specimens. This misclassification cannot be fully eliminated in current practice. Although MRI-EPE scoring is available before surgery, it remains a qualitative assessment that may be influenced by reader experience and image quality, potentially leading to variability in diagnostic interpretation. Therefore, incorporating objective and quantitative imaging biomarkers, such as histogram-derived parameters, may help overcome the limitations of conventional clinical indicators.

Histogram analysis provides multiple quantitative parameters capable of capturing subtle variations in voxel intensity distributions, enabling a more comprehensive evaluation of tumor heterogeneity (28). Because ADC is a well-recognized imaging biomarker reflecting the invasiveness and microstructural characteristics of prostate cancer, ADC histogram analysis has become increasingly important in PCa research (8, 11, 17). In this study, Minimum and TotalEnergy emerged as the key histogram features within the peritumoral regions. Minimum represents the voxel with the greatest restriction of water diffusion within a given region, typically corresponding to areas of increased cellular density and reduced extracellular space, and it was significantly lower in the EPE-positive group (9). TotalEnergy, defined as the sum of squared voxel intensities, was significantly elevated in the EPE-positive group and is particularly sensitive to signal dispersion and extreme values (29). Higher TotalEnergy values may reflect the coexistence of low-signal components, such as densely packed tumor cells or fibrosis, with high-signal components, including normal glandular tissue or oedema, indicating increased tissue heterogeneity.

The progression of EPE in prostate cancer is generally considered a gradual outward invasive process. The peritumoral region immediately adjacent to the tumor margin, particularly within 1 mm, is likely to represent the primary site of microscopic invasion. High-density infiltrative tumor cells within this area may significantly restrict water diffusion, resulting in lower ADC Minimum values. At the same time, the coexistence of infiltrative tumor tissue and adjacent normal structures may increase signal heterogeneity, thus elevating TotalEnergy. As the peritumoral distance expands to 2–3 mm, the density of infiltrating tumor cells likely decreases progressively, and higher ADC values from surrounding normal tissues dilute the extreme signal characteristics, resulting in reduced differences in Minimum and TotalEnergy between groups and a more homogeneous signal distribution. A gradual decline was also observed in diagnostic performance as the peritumoral ROI distance increased. This finding suggests that the invasive growth pattern of prostate cancer exhibits spatial heterogeneity and that the imaging information derived from the peritumoral microenvironment varies according to the distance from the tumor boundary. Zhou et al. (12) similarly reported that excessively large peritumoral regions may include substantial amounts of normal tissue, introducing nonspecific background signals and reducing model discriminative ability. These observations may provide useful guidance for designing peritumoral ROI boundaries in future EPE-related studies.

Forward stepwise logistic regression did not identify intratumoral parameters as dominant predictors in the multi-region histogram analysis. Moreover, the diagnostic performance of the 1-mm and 2-mm peritumoral models exceeded that of the intratumoral model. These findings suggest that peritumoral information reflecting the tumor–host interface may offer more specific discrimination than intratumoral characteristics alone. A possible explanation is that intratumoral histogram features mainly describe variations in cell density and necrosis distribution and are more strongly associated with tumor grading, such as the Gleason score (30), but may not adequately capture capsular invasion. Notably, SHAP analysis revealed that peri1_Minimum contributed most strongly to model prediction, whereas peri3_Minimum showed an opposite directional contribution. One possible explanation is that the peri_1mm region immediately adjacent to the tumor contains densely infiltrating tumor cells that severely restrict water diffusion, resulting in extremely low ADC values. In comparison, the peri_3mm region may contain fewer invasive tumor cells and more reactive stromal changes, such as fibrosis, oedema, or angiogenesis, resulting in relatively higher ADC values, although still lower than those of normal prostate tissue, which remains to be confirmed by pathological studies. If confirmed, an “inner low, outer relatively high” ADC gradient pattern might more accurately characterize the dynamic evolution of the peritumoral microenvironment during EPE progression than a single Minimum value alone. However, we acknowledge that this mechanistic interpretation requires independent pathological or functional validation in future studies.

Compared with previous studies, Chen et al. (9) reported that intratumoral ADC histogram parameters, including the 10th, 90th, mean, median, and minimum percentiles, were significantly lower in EPE-positive patients, findings consistent with our results. However, the best-performing parameter in their study was Median, with an AUC of 0.681. Similarly, Krishna et al. (31) identified Entropy as the most discriminative parameter, with an AUC of 0.76. In comparison, peri1_Minimum achieved the highest predictive value in our study, with an AUC of 0.825. Furthermore, Bai et al. (25) also demonstrated that peritumoral features outperform intratumoral features in EPE prediction, further supporting the incremental diagnostic value of the peritumoral microenvironment. However, their radiomics models performed worse than ours, which may be attributable to the use of wider peritumoral margins (3–12 mm). Inclusion of extensive surrounding normal tissue may dilute critical imaging features and reduce model sensitivity to early invasive changes. These findings further support the concept that the most clinically relevant imaging information for EPE prediction is concentrated within the immediate peritumoral microenvironment.

This study adopted a rigorous image standardization workflow and systematically segmented peritumoral regions into 1-, 2-, and 3-mm zones for comparative analysis. By restricting enrollment to PI-RADS 4–5 lesions, we minimized confounding from indolent disease and improved the clinical relevance of the model for high-risk prostate cancer populations. Unlike high-dimensional radiomics approaches, this study utilized histogram parameters with relatively low dimensionality, improving model simplicity, robustness, and interpretability while maintaining strong diagnostic performance. The combined model generated continuous probability outputs that could be integrated into a nomogram for individualized EPE risk estimation, providing practical support for clinical decision-making.

Nevertheless, bootstrap internal validation was performed on the final fixed model after feature selection, without repeating the feature selection process within each bootstrap iteration. This approach may underestimate the true optimism of model performance. To address this, we assembled an additional temporal validation cohort from recent clinical practice at the same institution and applied the pre-trained models directly to this unseen dataset. In this temporal validation cohort, the combined model achieved an AUC of 0.861, which was numerically higher than that of the clinical model (0.818) and the histogram model (0.796). The DeLong test did not show a statistically significant difference between the combined and clinical models (p > 0.05). Interestingly, the difference in ISUP grade between EPE-positive and EPE-negative patients did not reach statistical significance, likely due to the smaller sample size and temporal variations in patient composition. However, compared with the clinical model, the combined model incorporating peritumoral ADC features showed a continuous NRI of 0.7286 (95% CI: 0.0971˜1.3601, p = 0.024), and an IDI of 0.0919 (p = 0.053), indicating a positive trend toward improved overall performance, although it did not reach the conventional significance threshold, possibly due to the limited sample size of the validation cohort. It is important to clarify that continuous NRI quantifies the net direction of risk re-ranking across all patients. While AUC primarily reflects the overall ranking concordance of a model and may be relatively insensitive to modest but clinically relevant shifts in risk estimates. The significant NRI observed in the temporal validation, together with the favorable decision curve analysis results, supports the hypothesis that peritumoral ADC histogram features may provide complementary, clinically actionable information beyond conventional clinical indicators (ISUP grade and MRI-EPE score). Nevertheless, these findings must be interpreted cautiously given the non-significant AUC difference between the two models, and require validation in larger, multicenter cohorts to confirm their clinical utility.

These findings suggest that the combined model not only shows reasonable generalizability across different time periods but may also offer incremental value primarily in terms of improved risk stratification for individual patients. We acknowledge that the non-significant AUC difference indicates that the combined model does not fundamentally outperform the clinical model in ranking ability; nevertheless, the significant NRI suggests that it may enhance the precision of clinical decision-making. Additionally, the combined model, which integrates clinical variables (ISUP grade and EPE status) with the histogram features, achieved the lowest Brier score and the calibration slope closest to 1. This improvement suggests that adding clinically relevant covariates helps to attenuate the systematic over-prediction and enhances the overall accuracy of the predicted probabilities. The Brier score of the combined model (0.150) was approximately 7% lower than that of the clinical model alone (0.161), supporting the added value of a multimodality approach. The value of integrating complementary information sources is increasingly recognized across medical imaging. Xu et al. (32) and Archibald et al. (33) have respectively demonstrated this paradigm in neurodegenerative diseases and multimodality oncologic imaging. Although our study focuses on ADC histogram analysis rather than nuclear medicine, the underlying principle—that combining clinical, morphological, and quantitative microstructural information improves diagnostic performance—is consistent with these broader trends. Our findings suggest that peritumoral ADC features, when combined with clinical predictors, may provide a form of multiparametric risk stratification analogous to the multimodality approaches in other fields.

Our findings may hold implications for radiotherapy target volume delineation in prostate cancer. In current practice, clinical target volume (CTV) expansion often applies a uniform margin without adjusting for individual EPE risk (34). The ADC gradient reflected by peri1_Minimum and peri3_Minimum may serve as an objective imaging surrogate to guide patient-specific CTV margins. The presence of Peri1_Minimum indicates that the tumor cells have densely infiltrated the 1 mm area surrounding the tumor. Such patients may require a more aggressive expansion of the boundary. The relative change of Peri3_Minimum reflects the depth of micro-infiltration, which can assist in determining whether it is necessary to further expand the CTV to cover potential microscopic lesions. In the era of MRI-guided adaptive radiotherapy (35) and dose-painting strategies, integrating ADC histogram parameters into treatment planning could enhance the therapeutic ratio by balancing tumor control probability with sparing of adjacent organs at risk. These hypotheses, however, warrant prospective validation in radiation-oncology cohorts.

This study has several limitations that should be acknowledged. First, this was a single-center retrospective study with a relatively small sample size. Therefore, the generalizability and robustness of the findings require further confirmation through large-scale multicenter prospective studies. Second, the dimensionality of histogram-derived features was relatively limited. Future studies could incorporate multiparametric MRI sequences and higher-order imaging biomarkers to enhance model performance further. Third, the reliance on semi-automated ROI delineation posed challenges in accurately measuring volumes due to the irregular contours of peritumoral ROI. Implementing fully automated deep learning segmentation in future studies could facilitate standardized volumetric assessments and better define the independent utility of peritumoral features. Fourth, in patients with multifocal lesions, the index lesion was defined by preoperative biopsy Gleason score and MRI diameter. However, biopsy sampling error—an inherent limitation—may cause discordance between the preoperatively defined index lesion and the final pathological findings. Fifth, although ADC maps were resampled to 1 × 1 × 1 mm³ to standardize peritumoral expansion and feature extraction, we acknowledge that resampling does not create true isotropic spatial resolution. Future studies employing high-resolution DWI with thinner slices would be valuable to confirm our findings. Notably, no tumors originating from the central zone or anterior fibromuscular stroma were identified in this cohort, limiting our ability to assess the model’s performance in these rare locations.

## 5 Conclusion

In conclusion, this study suggests that ADC-based peritumoral histogram features show promising diagnostic value for predicting EPE in PI-RADS 4–5 prostate cancer, with their predictive performance gradually decreasing as the peritumoral distance increases. In the independent temporal validation cohort, the combined model integrating the Minimum values from the 1-mm and 3-mm peritumoral regions with ISUP grade and MRI-EPE score demonstrated a significant continuous NRI of 0.7286 (*p* = 0.024), indicating that the addition of peritumoral ADC histogram features may provide complementary information to conventional clinical assessment and potentially improve risk stratification. From a radiotherapy planning perspective, the peri1_Minimum and peri3_Minimum features may offer objective imaging surrogates for guiding individualized clinical target volume (CTV) expansion in patients at high risk of EPE, with potential applications in both external beam radiotherapy and brachytherapy. However, given the non-significant difference in AUC between the combined and clinical models (*p* > 0.05) and the borderline significance of the IDI, these findings should be interpreted as hypothesis-generating rather than definitive evidence of clinical superiority. Notably, our findings require further validation in larger, preferably multicenter, prospective cohorts before clinical implementation. Overall, these findings offer imaging-based insights into the potential role of the peritumoral microenvironment in tumor invasion and may provide a useful reference framework for individualized treatment decisions in patients with high-risk prostate cancer.

## Data Availability

The original contributions presented in the study are included in the article/**Supplementary Material**. Further inquiries can be directed to the corresponding author.
