# Supplementary material for: Diagnostic value of whole-tumor ADC histogram parameters combined with ISUP grade and MRI-EPE score for predicting extracapsular extension in PI-RADS 4–5 prostate cancer
Source: Front Oncol. 2026 Aug 17;16:1886143. doi: 10.3389/fonc.2026.1886143 (PMC13526556; doi:10.3389/fonc.2026.1886143)
Supplement: Supplementary file 1 [file DataSheet1.docx]

Supplementary TABLE S1. Baseline data and tumour MRI characteristics between groups in the temporal validation cohort

| Characteristics | EPE- (n=20) | EPE+ (n=14) | *p* value |
| --- | --- | --- | --- |
| age (year) | 70.90 ± 6.47 | 72.14 ± 6.96 | 0.602^a^ |
| maximum diameter (cm) | 1.25 (0.90, 1.63) | 3.15 (2.43, 4.20) | **<0.001**^b^ |
| tPSA（ng/ml) | 8.36 (5.17, 10.16) | 67.73 (26.91, 107.15) | **<0.001**^b^ |
| fPSA（ng/ml) | 0.68 (0.39, 1.31) | 3.19 (1.54, 15.43) | **<0.001**^b^ |
| volume (cm3) | 40.65 (25.93, 51.33) | 51.90 (35.98, 68.00) | 0.128^b^ |
| PSAD | 0.25 (0.19, 0.30) | 0.90 (0.63, 1.30) | **<0.001**^b^ |
| ISUP grade n (%) |  |  | 0.198^c^ |
| 1 | 5 (25.00) | 0 (0.00) |  |
| 2 | 5 (25.00) | 2 (14.29) |  |
| 3 | 6 (30.00) | 6 (42.86) |  |
| 4 | 2 (10.00) | 2 (14.29) |  |
| 5 | 2 (10.00) | 4 (28.57) |  |
| location n (%) |  |  | **0.003**^c^ |
| bilateral zone | 2 (10.00) | 9 (64.29) |  |
| peripheral zone | 9 (45.00) | 4 (28.57) |  |
| transition zone | 9 (45.00) | 1 (7.14) |  |
| PI-RADS score n (%) |  |  | **<0.001**^c^ |
| 4 | 12 (60.00) | 0 (0.00) |  |
| 5 | 8 (40.00) | 14 (100.00) |  |
| MRI-EPE score n (%) |  |  | **0.001**^c^ |
| 0 | 6 (30.00) | 0 (0.00) |  |
| 1 | 12 (60.00) | 5 (35.71) |  |
| 2 | 2 (10.00) | 4 (28.57) |  |
| 3 | 0 (0.00) | 5 (35.71) |  |

EPE-, EPE-negative; EPE+, EPE-positive; ^a^Comparisons were performed by independent samples t-test; ^b^Comparisons were performed by Mann−Whitney U test; ^c^Comparisons were performed by χ² test; p values in bold denote statistical significance.

Supplementary TABLE S2. Model specifications: variables, regression coefficients, intercept, odds ratios, 95% confidence intervals, and calculation formulas for each predictive model

|  | Variable | B | Standard Error (SE) | Odds Ratio (OR) | 95% CI | *p* value |
| --- | --- | --- | --- | --- | --- | --- |
| clinical model | ISUP grade | 0.994 | 0.296 | 2.701 | 1.551-4.828 | ＜0.001 |
|  | EPE score | 1.694 | 0.427 | 5.441 | 2.357-12.558 | ＜0.001 |
| histogram model | Peri1_Minimum | -4.209 | 1.098 | 0.015 | 0.002-0.128 | ＜0.001 |
|  | Peri3_Minimum | 2.398 | 0.890 | 11.006 | 1.924-62.950 | 0.007 |
| combined model | clinical model predicted probability | 4.547 | 1.084 | 94.382 | 11.271-792.360 | ＜0.001 |
|  | histogram model predicted probability | 2.983 | 1.184 | 19.744 | 1.938-201.180 | 0.012 |

Supplementary TABLE S3. Comparison of original histogram parameters of different regions of interest groups in the training cohort

| Segmentation region | Parameters | EPE- (n=51) | EPE+ (n=51) | *p* value |
| --- | --- | --- | --- | --- |
| intratumor | Intra_90Percentile | 820.81 ± 126.87 | 703.91 ± 103.01 | **<0.001**^a^ |
|  | Intra_TotalEnergy | 525818108.64 (252199249.68, 1398619292.50) | 1542820385.75 (725179317.25, 4092827725.37) | **<0.001**^b^ |
| peritumoral 1 mm | Peri1_Minimum | 591.89 ± 122.83 | 435.32 ± 126.03 | **<0.001**^a^ |
|  | Peri1_TotalEnergy | 494276381.07 (268915795.34, 1032758825.41) | 1400178376.54 (635400227.89, 2575017763.43) | **<0.001**^b^ |
| peritumoral 2 mm | Peri2_Minimum | 576.88 ± 131.24 | 434.37 ± 117.95 | **<0.001**^a^ |
|  | Peri2_TotalEnergy | 1191918675.28 (690399149.95, 2244460271.95) | 3387158554.20 (1820042350.41, 5926069521.24) | **<0.001**^b^ |
| peritumoral 3 mm | Peri3_Minimum | 578.66 (506.62, 644.04) | 428.22 (351.26, 510.49) | **<0.001**^b^ |
|  | Peri3_TotalEnergy | 2103554539.30 (1479043303.70, 5071885341.98) | 5916828578.53 (3104041704.29, 11152080536.43) | **<0.001**^b^ |
| Intra+Peri | Peri1_Minimum | 591.89 ± 122.83 | 435.32 ± 126.03 | **<0.001**^a^ |
|  | Peri3_Minimum | 578.66 (506.62, 644.04) | 428.22 (351.26, 510.49) | **<0.001**^b^ |

Intra_, intratumoral; Intra+Peri, Intratumoral combined with peritumoral; Peri1_, peritumoral 1 mm; Peri2_, peritumoral 2 mm; Peri3_, peritumoral 3 mm; ^a^Comparisons were performed by independent samples t-test; ^b^Comparisons were performed by Mann−Whitney U test; p values in bold denote statistical significance.

Supplementary TABLE S4. Calibration metrics for the three models in the temporal validation cohort.

| Model | Calibration slope (β) (95% CI) | Calibration‑in‑the‑large (α) (95% CI) | O/E ratio (95% CI) | Brier score (95% CI) |
| --- | --- | --- | --- | --- |
| clinical model | 0.682  (0.248, 1.117) | −0.283  (−1.324, 0.757) | 0.932  (0.558, 1.342) | 0.161  (0.069, 0.253) |
| histogram model | 0.642  (0.151, 1.133) | −0.828  (−1.729, 0.074) | 0.778  (0.466, 1.120) | 0.203  (0.120, 0.286) |
| combined model | 0.710   (0.273, 1.148) | −0.505  (−1.550, 0.541) | 0.885  (0.530, 1.275) | 0.150   (0.057, 0.243) |

Supplementary FIGURE S1

Development and interpretation of a nomogram for individualized prediction of extraprostatic extension(EPE) . This nomogram was constructed based on a combined model integrating clinical and radiomic features. The x-axis represents the contribution of each variable to the linear predictor. To use this nomogram, locate the value of each predictor on its corresponding scale and draw a vertical line upwards to the "Points" axis to assign a specific score. The sum of these individual scores yields the "Total Points." Finally, draw a vertical line downwards from the total score to the "EPE Predicted Probability" axis to estimate the individual risk of EPE for a specific patient.
